# POLYPHARMACY AND THE SYMPTOM EXPERIENCE OF OLDER ADULTS WITH AND WITHOUT ALZHEIMER’S DISEASE AND RELATED DEMENTIAS

**DOI:** 10.1093/geroni/igad104.1368

**Published:** 2023-12-21

**Authors:** Martha Coates, Leslie McClure, Margaret Finley, Justine Sefcik, Laura Gitlin, Rose Ann DiMaria-Ghalili

**Affiliations:** Drexel University, Philadelphia, Pennsylvania, United States; Drexel University, Philadelphia, Pennsylvania, United States; Drexel Univeresity, Philadelphia, Pennsylvania, United States; Drexel University College of Nursing and Health Professions, Philadelphia, Pennsylvania, United States; Drexel University, Philadelphia, Pennsylvania, United States; College of Nursing and Health Professions, Drexel University, Philadelphia, Pennsylvania, United States

## Abstract

Older adults, including those living with Alzheimer’s disease and related dementias (ADRD), experience polypharmacy (PPY) (>5 medications daily) due to multiple chronic conditions. High symptom burden in older adults with ADRD and multimorbidity is well known. However, there is a gap in knowledge regarding symptoms experienced by older adults with and without ADRD who experience PPY. To address this gap, we conducted a longitudinal case-control observational study utilizing Rounds 6 to 9 (2016 to 2019) of the National Health and Aging Trends Study (NHATS) datasets. PPY was categorized by the number of medications from Round 6. We categorized individuals into four groups based on presence of ADRD and/or PPY (PPY and ADRD, PPY only, ADRD only, no PPY/ADRD) (N=2052). We identified symptoms associated with PPY (hopelessness, anxiety, general pain, stomach pain, breathing problems, weight loss, balance problems, fatigue, insomnia) in each group over each time point. Among those with ADRD (11.8%), over half experienced PPY (52.3%). Older adults with PPY and ADRD experienced a mean of 4.85 symptoms over all time points compared to 3.52 (ADRD only), 2.79 (no PPY/ADRD) and 3.93 (PPY only) (p=<0.001). Symptom frequencies in the PPY and ADRD group remained higher than all other groups across all four time points. Understanding the impact of medications on older adult’s quality life is a goal of the 4Ms framework. Future research will examine the symptoms associated with PPY and ADRD, physical function and health outcomes to inform medication management for older adults with dementia.

